# Screening of Botanical Drugs against Lassa Virus Entry

**DOI:** 10.1128/JVI.02429-20

**Published:** 2021-03-25

**Authors:** Yang Liu, Jiao Guo, Junyuan Cao, Guangshun Zhang, Xiaoying Jia, Peilin Wang, Gengfu Xiao, Wei Wang

**Affiliations:** a State Key Laboratory of Virology, Wuhan Institute of Virology, Center for Biosafety Mega-Science, Chinese Academy of Sciences, Wuhan, China; b University of the Chinese Academy of Sciences, Beijing, China; c College of Life Sciences, Nankai University, Tianjin, China; University of Kentucky College of Medicine

**Keywords:** Lassa virus, glycoprotein complex, bergamottin, casticin, membrane fusion

## Abstract

Lassa virus (LASV) belongs to the Old World genus *Mammarenavirus* (family *Arenaviridae*). At present, there are no approved drugs or vaccines specific for LASV. In this study, high-throughput screening of a botanical drug library was performed against LASV entry using a pseudotype virus bearing the LASV envelope glycoprotein complex (GPC). Two compounds, bergamottin and casticin, were identified as micromolar-range inhibitors of LASV entry. A mechanistic study revealed that casticin inhibited LASV entry by blocking low-pH-induced membrane fusion. Analysis of adaptive mutants demonstrated that the F446L mutation, located in the transmembrane domain of GP2, conferred resistance to casticin. Furthermore, casticin antiviral activity extends to the New World pathogenic mammarenaviruses, and mutation of the conserved F446 also conferred resistance to casticin in these viruses. Unlike casticin, bergamottin showed little effect on LASV GPC-mediated membrane fusion, instead inhibiting LASV entry by blocking endocytic trafficking. Notably, both compounds showed inhibitory effects on authentic lymphocytic choriomeningitis virus. Our study shows that both casticin and bergamottin are candidates for LASV therapy and that the conserved F446 in LASV GPC is important in drug resistance in mammarenaviruses.

**IMPORTANCE** Currently, there is no approved therapy to treat Lassa fever (LASF). Our goal was to identify potential candidate molecules for LASF therapy. Herein, we screened a botanical drug library and identified two compounds, casticin and bergamottin, that inhibited LASV entry via different mechanisms.

## Abstract (precis)

Currently, there is no approved therapy to treat Lassa fever (LASF). Our goal was to identify potential candidate molecules for LASF therapy. Herein, we screened a botanical drug library and identified two compounds, casticin and bergamottin, that inhibited LASV entry via different mechanisms.

## INTRODUCTION

Lassa virus (LASV) is an enveloped, negative-sense, bisegmented RNA virus belonging to the genus *Mammarenavirus* (family *Arenaviridae*). Mammarenaviruses include 39 unique species currently recognized by the International Committee on Taxonomy of Viruses (1). The original classification of mammarenaviruses, based mainly on virus genetics, serology, antigenic properties, and geographical relationships, divided them into New World (NW) and Old World (OW) mammarenaviruses (2). OW LASV and Lujo virus (LUJV) as well as some NW mammarenaviruses, including the Junín virus (JUNV), Machupo virus (MACV), Guanarito virus (GTOV), Chapare virus (CHAPV), and Sabiá virus (SBAV), are known to cause severe hemorrhagic fever and are listed as biosafety level 4 (BSL4) agents (3, 4).

Mammarenavirus RNA genomes encode the viral polymerase, nucleoprotein, matrix protein (Z), and glycoprotein complex (GPC). GPC is synthesized as a polypeptide precursor that is sequentially cleaved by a signal peptidase and the cellular protease subtilisin kexin isozyme 1 (SKI-1)/site 1 protease (S1P) to generate the three subunits of the mature complex: the retained stable-signal peptide (SSP), the receptor-binding subunit GP1, and the membrane fusion subunit GP2 (2, 5–7). The three noncovalently bound subunits SSP, GP1, and GP2 form a (SSP/GP1/GP2)_3_ trimeric complex that is present at the surface of the mature virion and is essential for virus entry. The unusual retained SSP interacts with the membrane-proximal external region as well as the transmembrane domain of GP2 and thus stabilizes the prefusion conformation of GPC and provides an interface with the potential to be targeted by entry inhibitors (8–10).

Lassa hemorrhagic fever is an annual epidemic in West Africa and peaks during the dry seasons. In 2020, Nigeria faced a large outbreak, with 4,761 suspected and 1,006 confirmed cases as of 17 May, according to the Nigeria Centre for Disease Control. To date, no vaccines or specific antiviral agents against LASV are available. Therapeutic strategies are limited to the administration of ribavirin in the early course of the illness. To address this issue, we screened a botanical drug library of 1,058 compounds. Natural-product screening could increase the speed of discovery and development for treatment, provide a strategy for drug optimization, and facilitate the elucidation of the mechanism underlying the infection (11). All the compounds were extracted from botanicals with a purity of ≥98%. By using a pseudotype of LASV in BSL2 facilities, we determined that the compounds bergamottin and casticin inhibit LASV entry.

## RESULTS

### Bergamottin and casticin block LASV entry.

To construct viral systems to study viral entry in BSL2 laboratories and facilitate high-throughput screening, recombinant LASV (LASVrv) based on the vesicular stomatitis virus (VSV) backbone containing the gene for green fluorescent protein (GFP), as well as the LASV GPC gene, was constructed. LASVrv was replication competent at a titer of 1.6 × 10^7^ PFU/ml. A pseudotype of LASV (LASVpv) was also constructed with the VSV backbone, and the GPC gene was replaced with a luciferase gene. LASVpv was competent for a single round of viral entry and infection (9).

**FIG 1:**
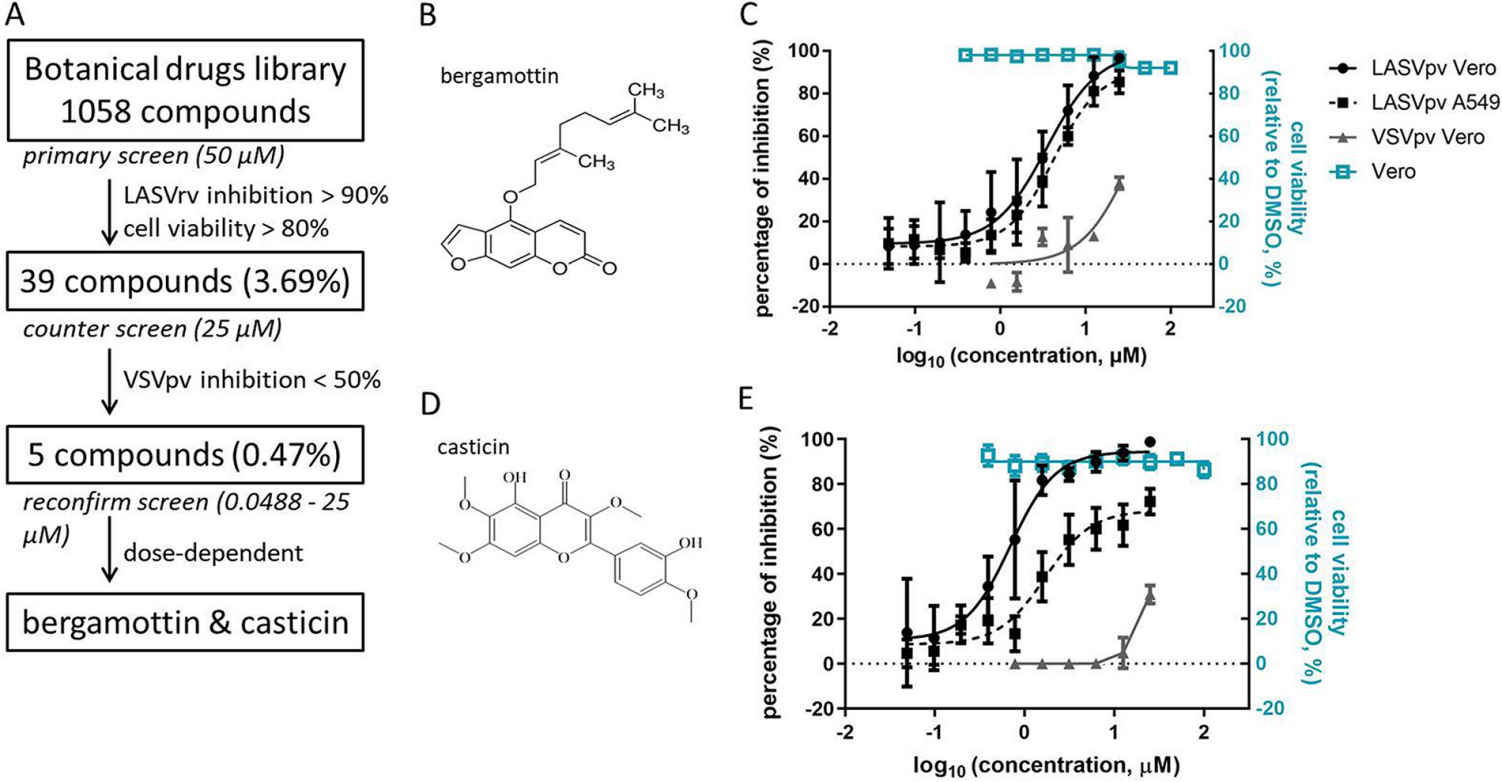
High-throughput screening (HTS) for inhibitors of Lassa virus (LASV) entry from a botanical drug library. (A) HTS assay flowchart. (B) Structure of bergamottin. (C) Dose-response curves of bergamottin. Cells were seeded at a density of 1 × 10^4^ cells per well in 96-well plates. After overnight incubation, cells were treated in duplicate with bergamottin at the indicated concentrations; 1 h later, cells were infected with LASVpv (MOI, 0.01) for 1 h. The infected cells were lysed 23 h later, and the luciferase activities were measured. Cell viability was evaluated using MTT assay. (D) Structure of casticin. (E) Dose-response curves of casticin. Data are means and standard deviation (SD) from six independent experiments.

As shown in Fig. 1A, bergamottin and casticin were selected as successful hits after three rounds of screening. Both compounds exhibited robust inhibition of LASVrv, whereas they had mild effects on VSV, suggesting that they inhibit LASV infection at the entry step. The 50% inhibitory concentrations (IC_50_s) of bergamottin and casticin against LASVpv on Vero cells were 3.615 and 0.6954 μM, respectively, while the values obtained for A549 cells were 4.300 and 1.696 μM, respectively, indicating that both compounds exhibit similar inhibitory effects on different cell types (Fig. 1C and E). As neither bergamottin nor casticin showed cytotoxicity at the highest tested concentration (100 μM), the selective index (SI; the ratio of the 50% cytotoxic concentration to the IC_50_) values were calculated to be >27.6 and >143.8, respectively. This suggests that both drugs are promising possibilities to combat Lassa fever (LASF).

### Casticin inhibits LASV GPC-mediated membrane fusion.

**FIG 2:**
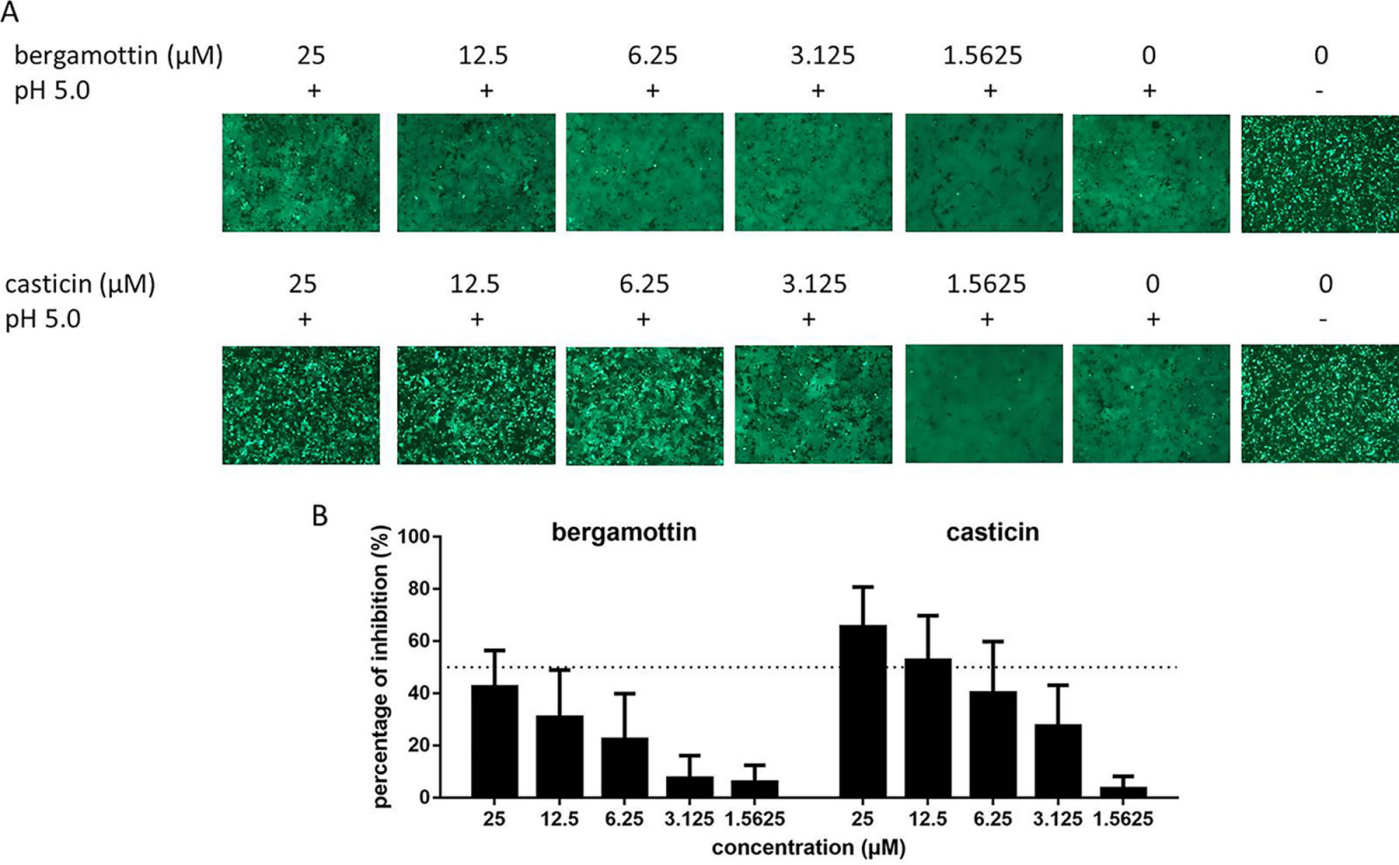
Bergamottin and casticin inhibited LASV GPC-mediated membrane fusion. (A) Qualitative evaluation of the inhibitory activities of bergamottin and casticin against membrane fusion. 293T cells were transfected with pCAGGS-LASV GPC; 24 h later, either the compounds or vehicle (DMSO) was added for 1 h followed by treatment with acidified (pH 5.0) DMEM for 15 min. The cells were then placed in neutral pH DMEM. Syncytium formation was visualized 1 h later using fluorescent microcopy. Images are representative fields from 4 or 5 independent experiments. (B) A dual-luciferase assay was used to quantitatively evaluate the inhibitory activities of bergamottin and casticin against membrane fusion. 293T cells transfected with both pCAGGS-LASV GPC and pCAG-T7 were cocultured at a ratio of 3:1 with targeted cells transfected with pT7EMCVLuc together with the pRL-CMV control vector. The cell fusion activity was quantitatively determined by measuring firefly luciferase activity expressed by pT7EMCVLuc and was standardized with Rluc activity expressed by pRL-CMV. Data are means and SD from 3 or 4 independent experiments.

As the unique SSP-GP2 interface plays critical roles in stabilizing the prefusion conformation of GPC and thus constitutes the “Achilles heel” targeted by most mammarenavirus entry inhibitors, the effects of bergamottin and casticin on LASV GPC-mediated membrane fusion were studied. As shown in Fig. 2A, casticin effectively blocked low-pH-triggered membrane fusion in a dose-dependent manner, whereas bergamottin showed little effect at all tested concentrations.

**FIG 3:**
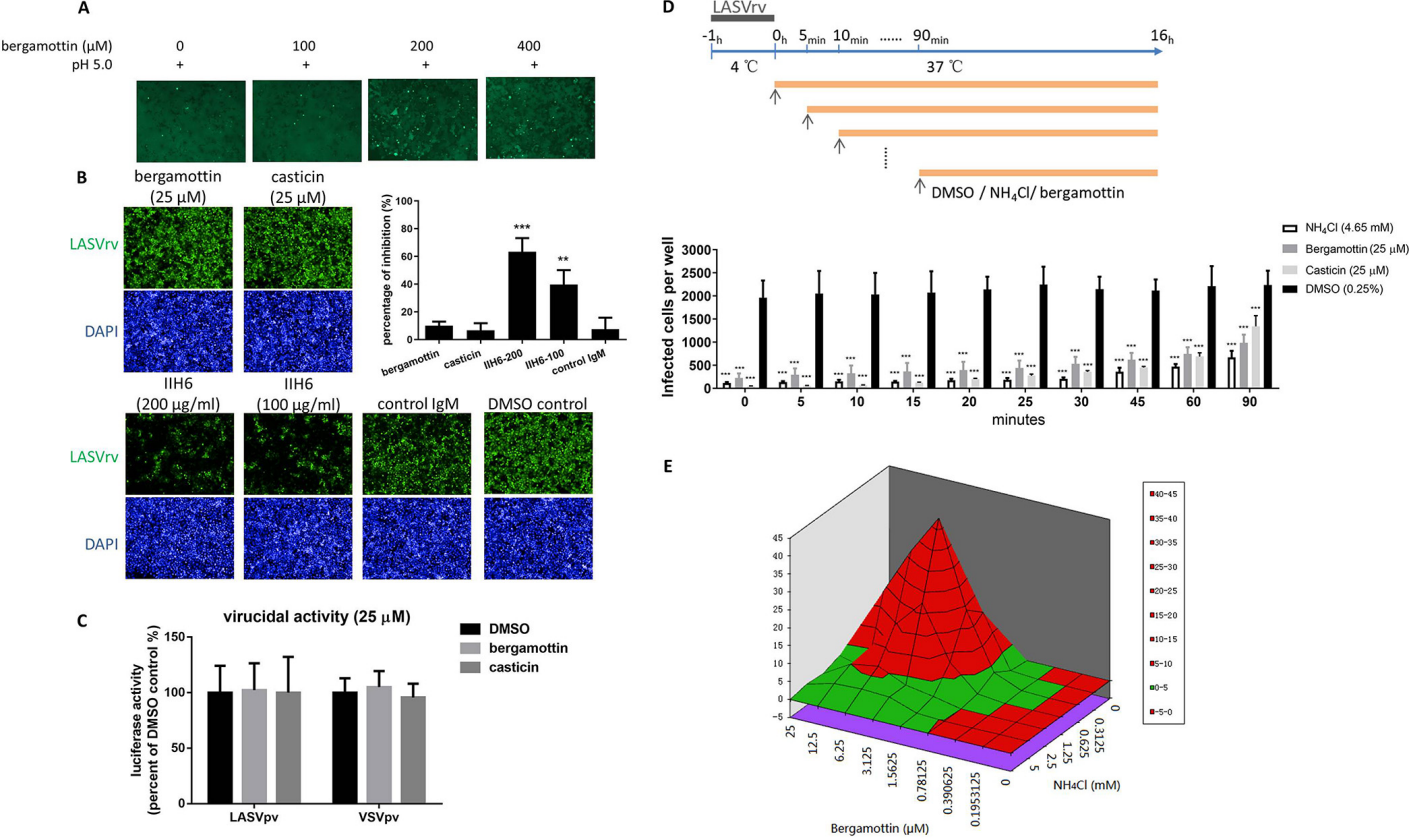
Bergamottin blocked LASVpv endocytic trafficking. (A) Effect of bergamottin on LASV GPC-mediated membrane fusion. 293T cells were transfected with pCAGGS-LASV GPC; 24 h later, bergamottin was added for 1 h followed by treatment with acidified (pH 5.0) DMEM for 15 min. The cells were then placed in neutral-pH DMEM. Syncytium formation was visualized 1 h later. Images are representative fields from 8 independent experiments. (B) Effects of bergamottin and casticin on LASVrv binding. A549 cells were preincubated with compounds or vehicle at 37°C for 1 h, followed by incubation with LASVrv (MOI, 1) in the presence or absence of compounds at 4°C for 1 h. The preincubation treatments with MAb IIH6 and an unrelated mouse IgM were conducted at 4°C for 2 h. After extensive washing with cold PBS, the GFP-positive cells were counted using an Operetta high-content imaging system 24 h later. Quantitative analysis of the percentage of inhibition was based on the numbers of positive cells. Inhibition was calculated as [(GFP_DMSO_/DAPI_DMSO_) − (GFP_tested_/DAPI_tested_)]/(GFP_DMSO_/DAPI_DMSO_). Error bars represent the SD from three independent experiments. ***, *P* < 0.001, and **, *P* < 0.01, compared to control IgM. (C) Assay of virucidal effects by bergamottin and casticin. Approximately 2 × 10^5^ PFU of LASVpv or VSVpv was incubated with compounds (25 μM) or vehicle at 37°C for 1 h; the mixture was then diluted 200-fold to infect Vero cells in a 96-well plate. Luciferase activity was determined 24 h later. (D) Time-of-addition assay. The experimental timeline is shown at the top. A549 cells were infected with LASVrv (MOI, 0.01) at 4°C for 1 h and then washed with PBS; the temperature was then increased to 37°C in the presence of bergamottin (25 μM), casticin (25 μM), and NH_4_Cl (4.65 mM) for the indicated times. (E) Drug-drug interactions of bergamottin with NH_4_Cl. Vero cells were infected with LASVrv (MOI, 0.01) and treated with various concentrations of bergamottin in combination with NH_4_Cl. Differential surface plots at the 95% confidence level were calculated and generated using MacSynergy II for the drug-drug interactions. Data are means and SD from 3 to 8 independent experiments. ***, *P* < 0.001, compared to DMSO.

To further quantitatively evaluate inhibition, fusion efficacy was determined using the dual-luciferase assay (9, 12–14). As shown in Fig. 2B, casticin exhibited a dose-dependent inhibition of GPC-mediated membrane fusion. Furthermore, the inhibition effect shown in the membrane fusion assay was similar to that obtained from the infection assay (Fig. 1E). Notably, casticin inhibited LASV GPC-mediated membrane fusion by ∼65% at 25 μM, while the inhibition by bergamottin was <50% at the same concentration. Bergamottin had no inhibitory effect on LASV GPC-mediated fusion at concentrations as high as 400 μM (Fig. 3A), suggesting that bergamottin blocks LASV entry via a different mechanism than casticin.

### Bergamottin blocks LASV endocytic trafficking.

To explore the mechanism underlying bergamottin inhibition, we further investigated the effects of both compounds on virus binding and endocytic trafficking. First, binding efficacy was evaluated in the absence and presence of the compounds in A549 cells, and the IIH6 antibody (a blocking antibody targeting the glycan epitope on α-dystroglycan [DG]) was used as the positive control (15, 16). As shown in Fig. 3B, IIH6 significantly blocked LASVrv transduction to the cell, while neither compound showed inhibitory effects, suggesting that neither bergamottin nor casticin interferes with the binding of LASV GP1 to the cell surface receptor.

Next, we tested whether both compounds could bind to LASV GPC and exert a virucidal effect. To this end, LASVpv (2 × 10^5^ PFU) was mixed with either compound at 25 μM for 1 h; the mixture was then diluted 200-fold to the noninhibitory concentration and added to the cells for 1 h (17). Luciferase activity was determined 23 h later. As shown in Fig. 3C, the luciferase activities observed in the compound-treated groups were similar to those in the dimethyl sulfoxide (DMSO) control group, suggesting that neither compound had a virucidal effect on LASVpv.

As bergamottin did not exert a virucidal effect or an inhibitory effect on the binding or fusion step, we hypothesized that it might inhibit endocytic trafficking. To address this, we quantitatively compared the inhibition of the endocytic efficiency by bergamottin with the acidification antagonist NH_4_Cl (18, 19). As shown in Fig. 3D, 1 h of incubation with LASVrv (multiplicity of infection [MOI] of 0.01) at 4°C led to approximately 2,000 cells being infected in a 96-well plate, while treatment with NH_4_Cl significantly decreased the number of infected cells to <200, especially within the first 30 min, in which LASV is supposed to undergo pH-dependent fusion (15, 20). Similar to the inhibitory effect of NH_4_Cl, bergamottin suppressed the early stage of LASVrv infection. These results suggest that bergamottin inhibits LASV entry by acting on the endocytic trafficking of the virus. Intriguingly, the combination of bergamottin and NH_4_Cl had significantly synergistic antiviral effects, suggesting that they might act on distinct targets in the endocytic trafficking step (Fig. 3E). Notably, we tried to select adaptive mutants by serially passaging LASVrv in the presence of bergamottin, and no mutant was obtained after 12 rounds of passage, which correlated with the results that bergamottin acted on the cellular endocytic pathway as opposed to targeting the virion.

We also conducted a time-of-addition assay with 25 μM casticin. At that concentration, casticin exerted an inhibitory effect similar to that of bergamottin and NH_4_Cl. Of note, casticin at lower concentrations (≤12.5 μM) showed little inhibition of VSVpv (Fig. 1E), which utilizes the endocytic pathway for entry (21). We suggest that high concentrations of casticin could exert nonspecific inhibition on the endocytic pathway. As casticin showed robust dose-dependent inhibition of GPC-mediated membrane fusion, it can be concluded that casticin inhibits LASV entry primarily by blocking fusion.

### The LASV GPC F446L mutation confers resistance to casticin.

**FIG 4:**
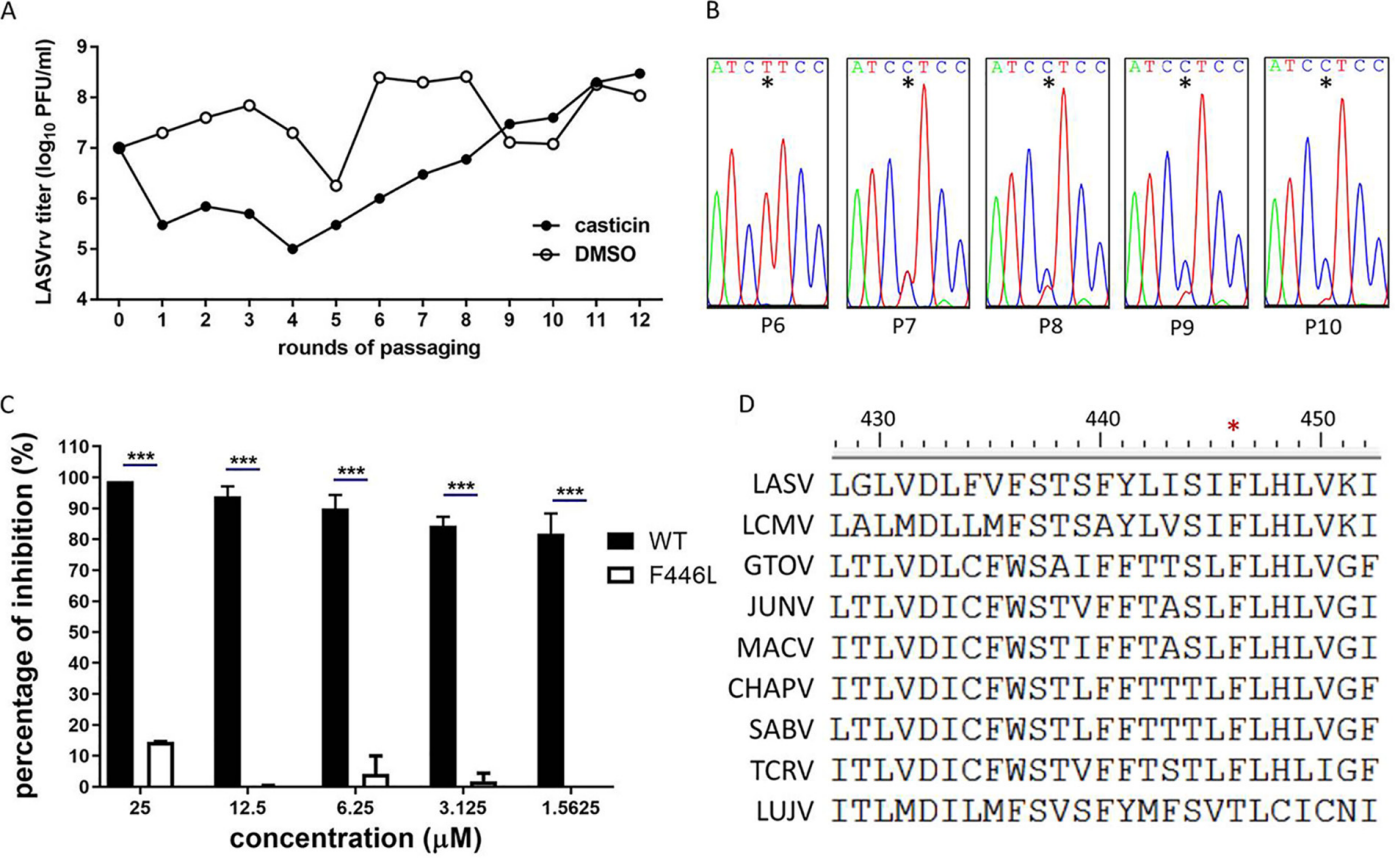
Selection of casticin-resistant LASVrv. (A) The adaptive mutant virus was selected by serially passaging LASVrv in the presence of 6.25 μM casticin. In a parallel experiment, LASVrv passaging in vehicle served as a control. (B) Sequencing chromatograms of viruses from P6 to P10. The asterisk highlights the emergence of the adaptive mutant. (C) Resistant activities to casticin of the WT and F446L LASVpv. Vero cells were treated with casticin at the indicated concentrations; 1 h later, WT or F446L LASVpv (MOI, 0.01) was added to the culture for 1 h. The infected cells were lysed 23 h later, and the luciferase activities were measured. Data are means and SD from 2 or 3 independent experiments. ***, *P* < 0.001. (D) Amino acid sequence alignment of the mammarenavirus transmembrane domain.

Since most arenavirus fusion inhibitors serve as direct antiviral agents by targeting the SSP-GP2 interface, we further investigated the viral targeting of casticin by selection for resistance mutations. As shown in Fig. 4A, after five rounds of passaging in the presence of 6.25 μM casticin (corresponding to an approximate IC_90_), LASVrv exhibited a steady increase of resistance to casticin which was absent in the DMSO control group. We sequenced the casticin-resistant LASVrvs from passage 6 (P6) to P10 without plaque purification. As shown in Fig. 4B, a resistant mutant with a change of a phenylalanine (TTC) to leucine (CTC) emerged at P7 and completed the substitution by P10. F446 is located at the transmembrane domain of GP2, which is highly conserved in mammarenaviruses (Fig. 4D). We introduced the F446L mutation into the GPC and generated a pseudotype of the mutant, LASV_F446L_pv. LASV_F446L_pv exhibited resistance to casticin. The inhibition efficiency against wild-type LASVpv infection reached ∼98% and ∼80% with casticin at 25 μM and 1.5625 μM, respectively, while only ∼15% inhibition of LASV_F446L_pv was observed with 25 μM casticin (Fig. 4C). Intriguingly, the F446 (corresponding to F438 in JUNV GPC) mutation has been reported to confer resistance to other structurally distinct compounds (22–25), suggesting that phenylalanine at this position might be accessible to different inhibitors and that the F446 mutation might contribute to the stabilization of GPC induced by the inhibitors.

### Casticin extends the antiviral spectrum to other mammarenaviruses.

**FIG 5:**
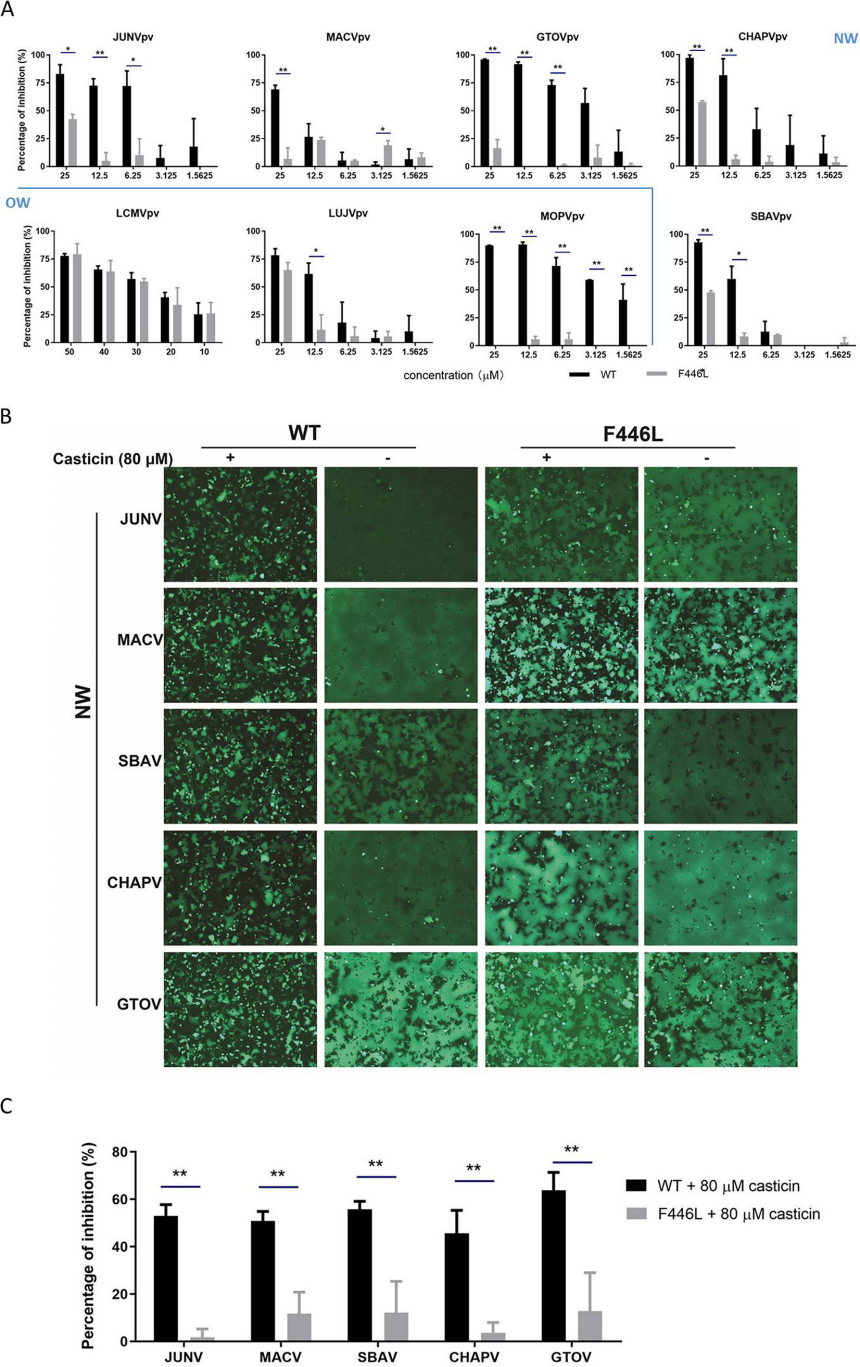
Antiviral effects of casticin on NW mammarenaviruses. (A) Vero cells were incubated in the absence and presence of casticin. After 1 h, the pseudotypes of JUNV, MACV, GTOV, CHAPV, SBAV, LCMV, LUJV, MOPV, and the corresponding F446L mutant viruses were added at an MOI of 0.1. The cell lysates were assessed for luciferase activity 23 h later. Data are means and SD from 3 independent experiments. *, *P* < 0.05; **, *P* < 0.01 (B) Casticin inhibited NW GPC-mediated membrane fusion, while the conserved phenylalanine mutation conferred resistance. 293T cells were transfected with pCAGGS-NW GPC; 24 h later, casticin (80 μM) was added for 1 h, followed by treatment with acidified (pH 5.0) DMEM for 15 min. Syncytium formation was visualized after 1 h. Images are representative fields from 3 independent experiments. (C) Quantitative analysis of the inhibitory effect of casticin on NW WT and F446L mutant GPC-mediated membrane fusion. 293T cells transfected with both pCAGGS-NW GPC (WT or F446L) and pCAG-T7 were cocultured at a ratio of 3:1 with targeted cells transfected with pT7EMCVLuc together with the pRL-CMV control vector. The cell fusion activity was quantitatively determined by measuring firefly luciferase activity expressed by pT7EMCVLuc and was standardized to Rluc activity expressed by pRL-CMV. Data are means and SD from 3 independent experiments. **, *P* < 0.01.

As F446 is highly conserved in mammarenaviruses (Fig. 4D), we asked whether casticin could inhibit entry of other arenaviruses. To address this, pseudotypes of pathogenic OW viruses (including the prototype lymphocytic choriomeningitis virus [LCMV], LUJV, and the most closely related Mopeia virus [MOPV]) and NW viruses (including JUNV, MACV, GTOV, SBAV, and CHAPV) were generated with the VSV backbone. As shown in Fig. 5, casticin effectively inhibited both NW and OW pseudotype virus infections in a dose-dependent manner. Moreover, we constructed the corresponding phenylalanine-to-leucine mutation in each virus pseudotype and tested the sensitivities of these mutant viruses to casticin. Notably, all the mutant NW pseudotype viruses, similar to LASV_F446L_pv, exhibited resistance to casticin, while the pathogenic OW viruses (LCMV and LUJV) bearing the leucine mutation were still sensitive to casticin. We hypothesize that casticin might inhibit LASV and NW mammarenavirus entry by a similar mechanism. To this end, we tested whether casticin inhibited membrane fusion caused by NW GPC. As shown in Fig. 5B and C, casticin at 80 μM inhibited NW GPC-mediated membrane fusion, while the corresponding F446L mutation conferred various degrees of resistance to casticin inhibition by GPC-mediated membrane fusion.

### Both casticin and bergamottin inhibit authentic LCMV infection.

**FIG 6:**
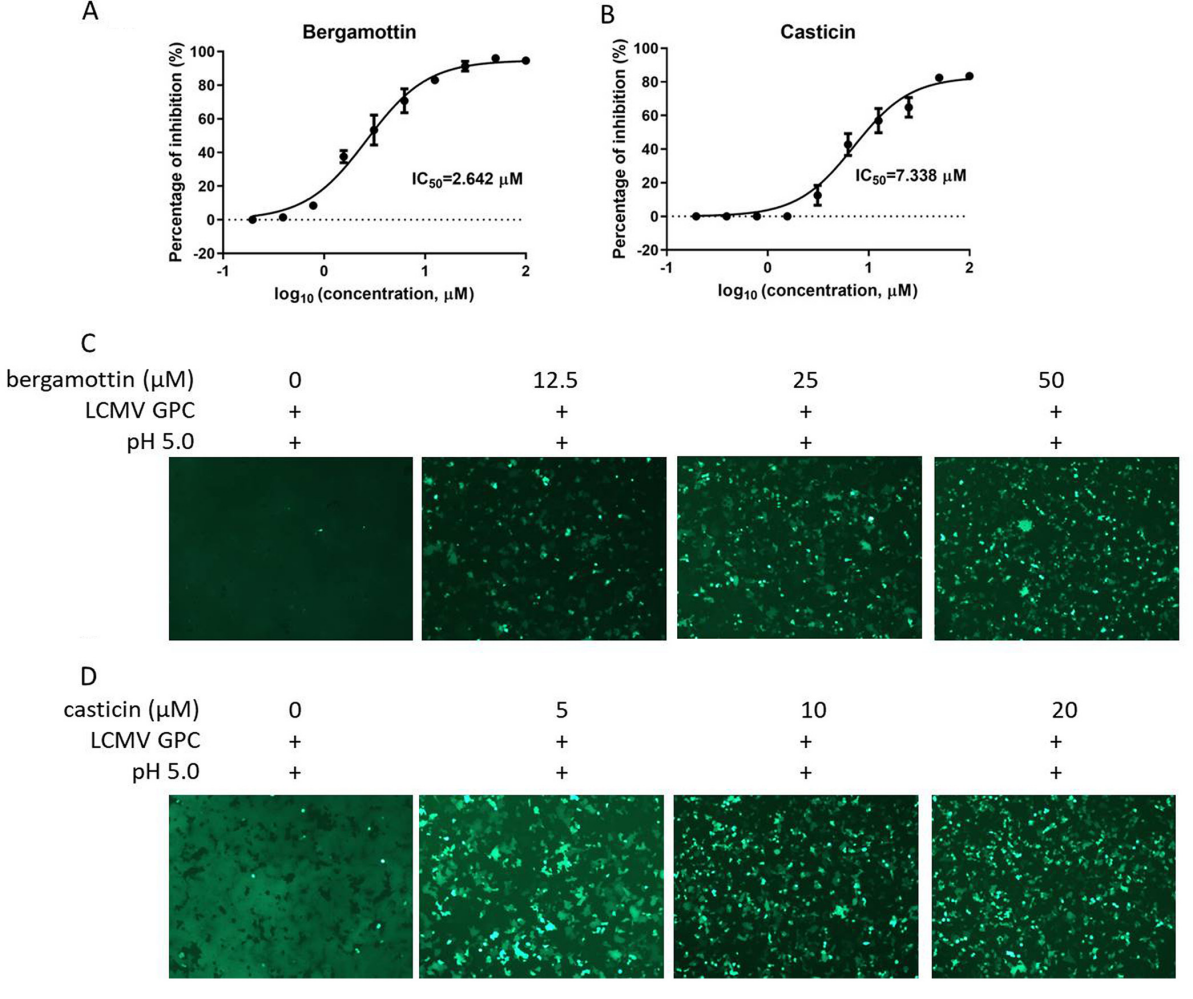
Inhibition of LCMV infection by bergamottin and casticin. Vero cells were treated in duplicate with bergamottin (A) and casticin (B), respectively. After 1 h, cells were infected with LCMV (MOI, 0.01) for 1 h. After 23 h, the inhibition is evaluated using qPCR with the primers for (5′-AGAATCCAGGTGGTTATTGCC-3′) and rev (5′-GTTGTAGTCAATTAGTCGCAGC-3′). Bergamottin (C) and casticin (D) inhibited LCMV GPC-mediated membrane fusion. 293T cells were transfected with pCAGGS-LCMV GPC; 24 h later, compounds or vehicle (DMSO) was added for 1 h followed by treatment with acidified (pH 5.0) DMEM for 15 min. The cells were then placed in neutral-pH DMEM. Syncytium formation was visualized 1 h later using fluorescent microcopy. Images are representative fields from 3 independent experiments.

To test the antiviral effects of both compounds on an authentic mammarenavirus, the BSL-2-compatible arenavirus, LCMV was utilized. As shown in Fig. 6A, bergamottin inhibited LCMV infection in a dose-dependent manner with an IC_50_ of 2.624 μM. Similarly, casticin inhibited LCMV infection with an IC_50_ of 7.388 μM (Fig. 6B), which was in line with casticin inhibition of LCMVpv infection (Fig. 5A). We further tested the inhibitory effects of both compounds against LCMV GPC-mediated membrane fusion. Intriguingly, both bergamottin and casticin showed inhibition against membrane fusion (Fig. 6C and D), suggesting that bergamottin might inhibit LCMV through a different mechanism than LASV (Fig. 3A). We attempted to obtain adaptive mutants of LCMV by serially passaging with increasing concentrations of bergamottin, but no mutants were observed in the entire genome.

### Both casticin and bergamottin inhibit the activity of LASV mini-genome.

**FIG 7:**
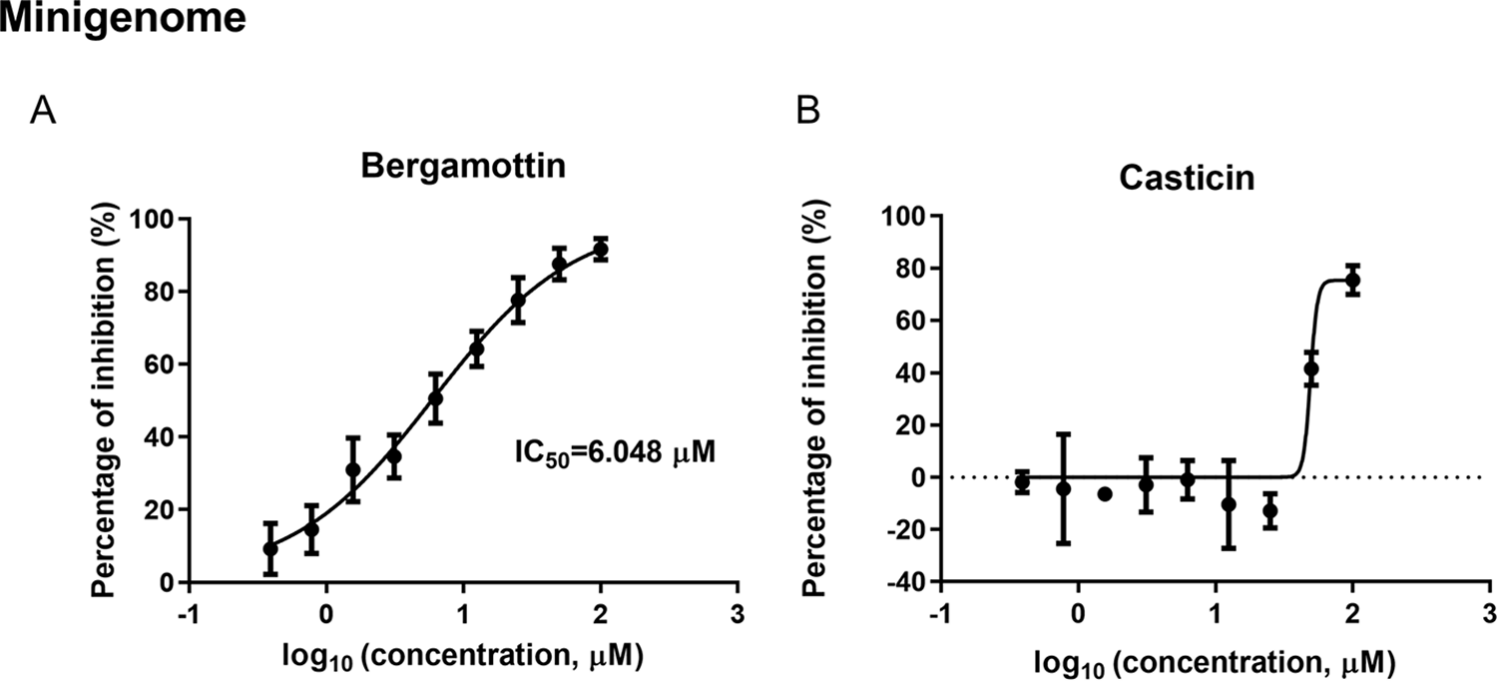
Bergamottin inhibits the replication of LASV. Vero cells in the 96-well plate were transfected with 30 ng pLASV-S (ZsG/gLuc), 10 ng pCAG-LASV-L, and 50 ng pCAG-LASV-NP. After 1 h, bergamottin and casticin were added at the indicated concentrations, and the luciferase activities were evaluated 48 h later.

To test whether casticin and bergamottin could inhibit the replication step of LASV, a LASV minigenome (MG) cell-based assay was conducted (26). As shown in Fig. 7, bergamottin exerted dose-dependent inhibition of LASV MG, whereas casticin showed a lessened inhibitory effect at concentrations above 50 μM, suggesting that bergamottin, and potentially casticin, might target RNA synthesis directed by the virus ribonucleoprotein complex.

## DISCUSSION

In this study, we screened a botanical drug library and identified two compounds, bergamottin and casticin, that blocked entry of pseudotype and recombinant LASV infection. Casticin is a constituent of *Vitex agnus-castus* seeds; natural products containing casticin have been extensively used to treat premenstrual syndrome (PMS) with few adverse effects (27, 28). Intravenous injection of 50 mg/kg in rats has been shown to lead to a maximum plasma concentration (*C*_max_) of ∼34 μM (29), which is far higher than the IC_50_ reported here. Notably, casticin has also been shown to inhibit CYP3A4 (27, 28), which is the most abundant CYP450 in the liver and intestines. Herein, we demonstrated that casticin inhibits LASVpv entry by acting as a direct antiviral agent (DAA). Casticin blocked the LASV GPC-mediated membrane fusion, and the viral target was the conserved phenylalanine located in the transmembrane domain of GP2. Furthermore, we found that the phenylalanine-to-leucine mutation at the corresponding site caused the NW pathogenic mammarenaviruses to confer resistance to casticin. Of note, when sequencing the resistant viruses, we observed only the mutant emerging in GPC. However, when we constructed the F446L mutant LASVpv *de novo* by introducing the mutant site into GPC and generated LASV_F446L_pv, resistance to casticin was conferred, indicating that the adaptive mutation in GPC made LASV resistant to casticin. Additionally, mutations at this position have been noted to confer resistance to distinct entry inhibitors (22–24). Whether the phenylalanine at this site was directly accessible to the compounds or worked allosterically to prevent viral entry or whether the mutation increased the stability of the glycoprotein requires further investigation.

We also identified bergamottin via high-throughput sequencing (HTS) because of its strong inhibition against LASV entry. Bergamottin is a natural furanocoumarin found principally in grapefruit, which has been shown to inhibit cytochrome P450 (CYP450) enzymes and thus affects the metabolism of many drugs (30, 31). Herein, we showed that bergamottin inhibits LASVpv entry by blocking viral endocytic trafficking, which seems to be independent of the effect on CYP450. Of note, we were unable to identify the specific target of bergamottin in anti-LASV inhibition. Although bergamottin showed a similar time course of inhibition to NH_4_Cl, it might act via a different mechanism from blocking the endosomal acidification, because the combination of bergamottin and NH_4_Cl had significantly synergistic antiviral effects. Both NW and OW arenaviruses utilize the endocytic pathway for entry. Pathogenic NW arenaviruses use TfR1 as the primary receptor and consistently engage in clathrin-mediated endocytosis, in which the gradually lower pH leads to endosome maturation (32). In contrast to NW, entry by the OW arenaviruses bypasses the early endocytic pathway and is instead initiated by binding to the primary receptor α-DG (33) and transported with the multivesicular bodies to the late endosome (20, 34). Notably, the OW species LUJV utilizes NRP2 as the primary receptor that is needed to switch the receptor to CD63 to complete productive entry (35, 36). Similarly, LASV undergoes receptor switching and engages LAMP1 in the late endosome for efficient fusion (15, 37). As both the NW and OW mammarenaviruses use receptor-mediated endocytosis to enter cells, bergamottin is thought to inhibit infections by blocking endocytic trafficking. Moreover, combination of bergamottin with other inhibitors might offer benefits beyond a single monotherapy.

In total, this study identified bergamottin and casticin as LASV entry inhibitors, highlighting two compounds derived from natural products that inhibit by different mechanisms. This study also highlighted the role of F446 in the SSP-GP2 interface in drug resistance in mammarenaviruses and identified the broad-spectrum antiviral effects of casticin.

## MATERIALS AND METHODS

### Cells and viruses.

BHK-21, BSR-T7, HEK 293T, Vero, HeLa, and A549 cells were cultured in Dulbecco’s modified Eagle’s medium (DMEM; HyClone, Logan, UT, USA) supplemented with 10% fetal bovine serum (Gibco, Grand Island, NY, USA).

The pseudotype VSV bearing the LASV GPC (Josiah strain, GenBank accession number HQ688673.1), LCMV (Armstrong strain; AY847350.1), LUJV (NC_012776.1), MOPV (AY772170.1), GTOV (NC_005077.1), JUNV (XJ13 strain; NC_005081.1), MACV (Carvallo strain; NC_005078.1), SBAV (U41071.1), and CHAPV (NC_010562.1) were generated as previously reported (38, 39). 293T cells transfected with pCAGGSGPC were infected at an MOI of 0.1 for 2 h with pseudotype VSV (described below) in which the G gene was replaced with a luciferase gene. The culture supernatants were harvested 24 h later, centrifuged to remove cell debris, and stored at −80°C. The recombinant VSV expressing LASV GPC was generated as described previously (9, 39, 40). The plasmid used for constructing the recombinant virus was pVSVΔG-eGFP (where eGFP is enhanced green fluorescent protein) (plasmid 31842; Addgene, Watertown, MA, USA). GPC was cloned into the ΔG site, and the construct was designated pVSVΔG-eGFP-GPC. BHK-21 cells grown in 6-well plates were infected with a recombinant vaccinia virus (vTF7-3) encoding T7 RNA polymerase at an MOI of 5. After 45 min, cells were transfected with 11 μg of mixed plasmids with a 5:3:5:8:1 ratio of pVSVΔG-eGFP-GPC (pVSVΔG-Rluc for generating pseudotype VSV), pBS-N, pBS-P, pBS-G, and pBS-L, respectively. After 48 h, the supernatants were filtered to remove the vaccinia virus and inoculated into BHK-21 cells that had been transfected with pCAGGS-VSV G 24 h previously. The pseudotype and recombinant viruses bearing LASV GPC were designated LASVpv and LASVrv, respectively. The titer of pseudotype virus was measured by infecting BHK-21 cells previously transfected with pCAGGS-VSV G and determined by plaque assay 24 h postinfection. The titer of the recombinant virus was determined by plaque assay. The titers of LASVpv and LASVrv were 3 × 10^7^/ml and 1.6 × 10^7^/ml, respectively.

Plasmid-based virus rescue of LCMV was carried out as previously reported (41). LCMV clone 13 genome RNA L (DQ361066) and S (DQ361065) segments were used to construct the plasmids pT7-LCMV-L and pT7-LCMV-S, respectively. BSR-T7 cells were transfected with the mixed plasmids of pCAGGS-LCMV-NP (300 ng), pCAGGS-LCMV-L (600 ng), pT7-LCMV-L (600 ng), and pT7-LCMV-S (300 ng). The supernatant was collected 72 h later and inoculated into BHK-21 cells for amplification. The titer of LCMV was determined to be was 1 × 10^7^/ml by plaque assay.

### Optimization of HTS assay conditions.

Cell density and MOI were optimized at 1 × 10^4^ cells and 1 × 10^2^ PFU per 96-well plate, respectively, as previously described (9, 42). Methyl-beta-cyclodextrin (MβCD; 2 mM) and 0.5% DMSO were used as the positive and negative controls, respectively. The signal-to-basal (S/B) ratio, coefficient of variation (CV), and Z′ factor were determined as 14,269, 1.64%, and 0.84, respectively.

### HTS assay of the botanical drug library.

A library of 1,058 botanical drug compounds was purchased from Weikeqi Biotech (Sichuan, China). The compounds were stored as 10 mM stock solutions until use. HTS was carried out as shown in Fig. 1A. Vero cells were treated in duplicate with the compounds (50 μM); 1 h later, cells were infected with LASVrv (MOI = 0.1) for 1 h. After 23 h, cells were fixed with 4% paraformaldehyde and stained with 4′,6-diamidino-2-phenylindole (DAPI; Sigma-Aldrich, St. Louis, MO, USA). Nine fields per well were imaged on an Operetta high-content imaging system (PerkinElmer, Waltham, MA, USA), and the percentages of infected and DAPI-positive cells were calculated using Harmony 3.5 (PerkinElmer). Cell viability was evaluated using a 3-(4,5-dimethyl-2-thiazolyl)-2,5-diphenyl-2H-tetrazolium bromide (MTT) assay. To determine the IC_50_, LASVpv at an MOI of 0.1 was used utilizing the timeline described above. Luciferase activity was measured using the *Renilla* luciferase (Rluc) assay system (Promega, Madison, WI, USA), and IC_50_ was calculated using GraphPad Prism 6.

### Membrane fusion assay.

293T cells cotransfected with pCAGGS-GPC and pEGFP-N1 were treated with either compounds or vehicle (DMSO) for 1 h, followed by incubation for 15 min with acidified (pH 5.0) medium. The cells were then placed in neutral medium, and syncytium formation was visualized 1 h later.

For quantification of the luciferase-based fusion assay, 293T cells in 24-well plates were transfected with both pCAGGS-GPC (0.25 μg) and plasmids expressing T7 RNA polymerase (pCAGT7, 0.25 μg) and cocultured at a ratio of 3:1 with targeted cells transfected with pT7EMCVLuc (2.5 μg per well in 6-well plates) and 0.1 μg pRL-CMV (plasmids used in this assay were kindly provided by Yoshiharu Matsuura, Osaka University, Osaka, Japan). After 12 h, compound treatment and pH induction were conducted as described above. Cell fusion activity was quantitatively determined after 24 h by measuring firefly luciferase activity expressed by pT7EMCVLuc and was standardized with Rluc activity expressed by pRL-CMV using the Dual-Glo luciferase assay (Promega) (9, 12–14).

### IIH6 inhibition assay.

A549 cells were pretreated with either 25 μM bergamottin or 25 μM casticin at 37°C for 1 h. As a control, A549 cells were pretreated either with IIH6 (sc-53987; Santa Cruz Biotechnology, Dallas, TX, USA) or a control IgM at 4°C for 2 h (15, 16). The cells were then transferred to ice, and LASVrv (MOI = 1) was added for 1 h. After three washes with cold phosphate-buffered saline (PBS), the cells were incubated at 37°C for 24 h. GFP-positive cells were counted using an Operetta high-content imaging system.

### Virucidal assay.

To study virucidal effects, approximately 2 × 10^5^ PFU of either LASVpv or VSVpv was incubated with compound (25 μM) or vehicle at 37°C for 1 h. The mixture was diluted 200-fold to a noninhibitory concentration to infect Vero cells in a 96-cell plate. Luciferase activity was determined 24 h later, as described above.

### Time-of-addition assay.

The experimental timeline is shown in Fig. 3D. At −1 h, A549 cells were infected with LASVrv (MOI = 0.01) at 4°C for 1 h and washed with PBS. The temperature was then increased to 37°C to synchronize the infections. The compounds were incubated with the cells for different time courses, as shown in Fig. 3D.

### Selection of adaptive mutants.

Drug-resistant viruses were generated by passaging LASVrv on Vero cells in the presence of 6.25 μM casticin. LASVrv was also passaged in the presence of 0.5% DMSO in parallel as a control. RNA from the resistant viruses was extracted using TRIzol (TaKaRa Bio, Inc., Kusatsu, Japan) and reverse transcribed using the PrimeScript RT reagent kit (TaKaRa). The GPC segment was amplified and sequenced as previously described (9). Mutant sites were introduced to LASVpv as previously described, and casticin sensitivities were determined by Rluc activity.

### MG assay.

The MG assay was conducted as previously reported (26). The LASV minigenome contains the antigenome 5′ and 3′ untranslated regions (UTRs) of the LASV S segment (Josiah strain; GenBank number HQ688673.1), in which the NP and GPC coding sequences have been replaced with those for ZsGreen (ZsG) and gLuc, respectively. Support plasmids encode LASV L and NP proteins. Vero cells in the 96-well plate were transfected with 90 ng of mixed plasmids with a 3:1:5 ratio of pLASV-S, pCAGGS-LASV-L, and pCAGGS-LASV-NP. After 1 h, the inhibitors were added at the indicated concentrations. Luciferase activity was evaluated 48 h later.
